# The role of the surface smear microbiome in the development of defective smear on surface-ripened red-smear cheese

**DOI:** 10.3934/microbiol.2018.4.622

**Published:** 2018-10-25

**Authors:** Jasmine S. Ritschard, Lea Amato, Yadhu Kumar, Britta Müller, Leo Meile, Markus Schuppler

**Affiliations:** 1 Laboratory of Food Microbiology, Institute of Food, Nutrition and Health, ETH Zurich, Schmelzbergstrasse 7, 8092 Zurich, Switzerland; 2 Laboratory of Food Biotechnology, Institute of Food, Nutrition and Health, ETH Zurich, Schmelzbergstrasse 7, 8092 Zurich, Switzerland; 3 Eurofins GATC Biotech AG, Jakob-Stadler-Platz 7, 78467 Konstanz, Germany

**Keywords:** microbiome, next-generation sequencing, surface ripened cheese, red-smear cheese, smear defect

## Abstract

The complex smear microbiota colonizing the surface of red-smear cheese fundamentally impacts the ripening process, appearance and shelf life of cheese. To decipher the prokaryotic composition of the cheese smear microbiome, the surface of a semi-hard surface ripened cheese was studied post-ripening by culture-based and culture-independent molecular approaches. The aim was to detect potential bacterial alterations in the composition of the cheese smear microbiota resulting from cheese storage in vacuum film-prepackaging, which is often accompanied by the development of a surface smear defect. Next-generation sequencing of amplified 16S rRNA gene fragments revealed an unexpected high diversity of a total of 132 different genera from the domains *Bacteria* and *Archaea* on the cheese surface. Beside typical smear organisms, our study revealed the presence of several microorganisms so far not associated with cheese, but related to milk, farm and cheese dairy environments. A 16S ribosomal RNA based analysis from total RNA identified the major metabolically active populations in the cheese surface smear as *Actinobacteria* of the genera *Corynebacterium*, *Brevibacterium*, *Brachybacterium* and *Agrococcus*. Comparison of data on a higher phylogenetic level revealed distinct differences in the composition of the cheese smear microbiome from the different samples. While the proportions of *Proteobacteria* and *Bacteroidetes* were increased in the smear of prepacked samples and in particular in defective smear, staphylococci showed an opposite trend and turned out to be strongly decreased in defective smear. In conclusion, next-generation sequencing of amplified 16S rRNA genes and 16S rRNA from total RNA extracts provided a much deeper insight into the bacterial composition of the cheese smear microbiota. The observed shifts in the microbial composition of samples from defect surface smear suggest that certain members of the *Proteobacteria* contribute to the observed negative organoleptic properties of the surface smear of cheese after prepacking in plastic foil.

## 1. Introduction

Cheese is a fermented milk based food product evolved 8000 years ago in the Middle East. Production of cheese was originally intended for bio-preservation of a nutritious food for human nourishment and developed nowadays to a premium food product comprising more than 1000 cheese varieties [1]. The varieties of surface-ripened cheeses have a long tradition in Europe and are diversified into bacterial surface-ripened red-smear cheeses and mold surface-ripened cheeses [2]–[4].

Bacterial surface-ripened red-smear cheeses such as Comté, Gruyère, Limburger or Tilsit are characterized by a high moisture content and a high ratio of surface area to volume [5]. These physical properties favor the influence of the microbial proteolytic and lipolytic activity on the intense aroma flavor formation, characteristic for this type of cheese [4],[6]. The viscous, red-orange smear developing on the cheese surface during ripening determines not only the organoleptic properties of these cheeses, but further serves as a protection against dryness and aroma loss [4],[7]. Moreover, fast growth of the surface microbiota during ripening and the inhibition properties of the inhabiting microorganisms protect the cheese from colonization by pathogenic foodborne bacteria or mycotoxin producing molds [8]–[10].

The surface-ripening of smear cheeses is a dynamic process and starts with the deacidification of the cheese surface by yeast, followed by a successive growth of characteristic red smear bacteria [2],[11],[12]. A smear layer composed of a multi-microbial species ecosystem develops naturally on the cheese surface when the young cheese is exposed to air at a high relative humidity (>95%) and suitable temperatures (13–15 °C) [2],[13]. To enhance the establishment of a stable surface microbiota and to suppress growth of molds, the surface is smeared periodically with brine during the ripening period [2],[6]. Naturally occurring indigenous smear microorganisms originate from the cheese-making environment such as cheese brines, raw milk and from the “house microbiota” in the ripening rooms or cheese-making premises, implying wooden shelves, vats, tap water and the room air [11],[14]–[17]. At the end of the ripening process mature red-smear cheese surface is mainly composed of Gram-positive bacteria and salt-tolerant yeast [2],[4].

For quality control and food safety issues the understanding of microbial composition, dynamics and interactions is essential to improve cheese taste, flavor, texture and safety [18]. Traditionally, the composition of the complex cheese surface microbiota was studied by culture-based methods followed either by phenotypic or genotypic identification of isolates [19]–[21]. Hitherto, less time-consuming molecular methods complement or replace cultivation dependent approaches [19],[21]. State-of-the-art molecular methods do not only allow to analyze the microbial diversity, but also monitor microbial dynamics and development over space and time [18],[22]. Application of next-generation sequencing technologies for high-throughput analyses opens new dimensions in the area of food microbiology by providing a deeper and more comprehensive insight into the community structure and the metabolic activity of microbial ecosystems [18],[21],[23],[24].

The aim of this study was to analyze the cheese surface smear microbiota in order to investigate the role of the smear bacteria in the development of cheese smear quality deterioration. Today, smear-ripened cheese is stored in vacuum film-prepackaging, which promotes the occurrence of a smear defect, appearing as a very humid, sticky and unpleasant off-flavored cheese surface [25]. Previous studies ruled out a contribution of yeast and pointed to alterations in the composition or the metabolic activity of smear bacteria due to changes in physico-chemical characteristics of the cheese smear after vacuum film-prepackaging [25],[26]. Consequently, the aim of this study was to perform an in-depth analysis to compare the bacterial composition and to identify metabolically active members of the surface microbiota in unpacked and vacuum film-prepacked cheese samples of differing qualities. For this purpose, the prokaryotic composition of the surface smear microbiota was analyzed by next-generation sequencing of multiplexed 16S rDNA amplicons. Furthermore, the metabolically active members of the cheese smear microbiota were determined by next-generation sequencing of 16S rRNA from total RNA extracts. The resulting 16S rDNA and rRNA data were compared with concurrent culture-based findings of the same smear samples.

## 2. Materials and methods

### 2.1. Cheese samples

The red-smear cheese investigated in this study was a Swiss semi-hard cheese variety with protection of origin (AOP), produced from raw milk from cows fed without silage. The use of additives in the cheese production is prohibited and the produced cheeses mature on spruce boards for at least 75 days at 13–14 °C and at relative air humidity of approximately 90%.

For analysis and comparison of the surface smear microbiome of cheeses differing in their smear quality all investigated samples derived from cheeses produced on the same day from the same milk and ripened in the same cheese cellar. All cheeses were treated equally until packaging at the end of the ripening period. Then the cheese portions were vacuum prepacked in gas- and watertight standard plastic film (Csf) by the manufacturer according to the standard procedure applied and subsequently stored at 8 °C during three weeks. A temperature of 8 °C was administered to mimic the storage conditions in a display cabinet or a domestic fridge [27],[28]. As a control, one cheese portion was not film-prepacked and further stored in the cheese cellar until sampling. During storage, the unpacked and one of the vacuum film-prepacked cheese portions (vacuum film-prepacked non-defective) retained a good quality surface smear (defined as a dry, non-smeary surface with a typical cheese odor). Other vacuum film-prepacked cheese portions developed a strong smear defect during storage (vacuum film-prepacked defective), which was characterized according to a previously described defect definition scheme [26] as a humid and smeary consistency with a very intensive off-odor pronounced in an animal-like flavor.

### 2.2. Culture-based analysis of cheese surface smear samples

Cheese surface smear samples from cheeses with differing smear quality were collected as previously described [25]. In brief, a rectangle of 8 cm^2^ size and 2–3 mm thickness (corresponding to 2.3 ± 0.2 g weight) was cut out of the cheese surface by a sterile knife. The cheese smear samples were diluted in 50 ml pre-warmed (45 °C) peptone solution (pH 7) composed of 1% (*w/v*) peptone from casein and 8.5% (*w/v*) NaCl (both Merck, Darmstadt, Germany) and thereafter homogenized during 4 min in a homogenizer (Colworth Stomacher 400; Bender & Hobein, Zurich, Switzerland). Colony forming units were determined by surface plating of serial dilutions on different non-selective and selective growth media. Total aerobic mesophilic and total anaerobic bacterial colony counts were determined on Tryptic Glucose Yeast Agar (TGYA; Biolife, Milano, Italy) supplemented with 1% (*w/v*) peptone from casein (Merck, Darmstadt, Germany), heterotrophic marine bacteria (halotolerant bacteria) on Marine Broth Agar (MB; Becton Dickinson AG, Allschwil, Switzerland), facultative anaerobic halophilic and alcaliphilic (FAHA) bacteria on Glucose Yeast Extract Peptone Beef Extract (GYPB) Agar [10], enterococci on KF *Streptococcus* Agar (KFS; Becton Dickinson AG, Allschwil, Switzerland), strictly anaerobic bacteria on Differential Reinforced Clostridial Medium (DRCM; Becton Dickinson AG, Allschwil, Switzerland) supplemented with 1 mg l^−1^ resazurin (Sigma-Aldrich, Steinheim, Germany) and 500 mg l^−1^ cysteine (VWR, Dietikon, Switzerland), and *Enterobacteriaceae* on Violet Red Bile Glucose Agar (VRBG; Biolife, Milano, Italy). The incubation properties were as follows: TGYA aerobic respectively anaerobic (3 days 30 °C aerobic respectively anaerobic incubation, followed by 7 days 22 °C room temperature incubation under day light), MB Agar (7 days 22 °C room temperature aerobic incubation under day light), GYPB Agar (4 days 30 °C anaerobic incubation), KFS Agar (3 days 42 °C aerobic incubation), DRCM Agar (4 days 22 °C strict anaerobic incubation in anaerobic chamber), VRBG Agar (1 day 37 °C aerobic incubation), PY Agar (3 days 30 °C aerobic incubation for yeast colony counts, respectively 6 days 30 °C aerobic incubation for mold colony counts). Colony counts were determined as weighted average.

### 2.3. Next-generation sequencing analysis of amplified 16S rRNA gene fragments

From each sample type (unpacked, vacuum film-prepacked non-defective and vacuum film-prepacked defective) total DNA and total RNA was extracted for subsequent next-generation sequencing analysis.

DNA was extracted from 350 µl of the previously prepared cheese smear raw extract by administering the GenEluteTM Bacterial Genomic DNA Kit (Sigma-Aldrich Chemie GmbH, Steinheim, Germany) according to the manufacturer's instruction with the following modifications: The heated incubation times were doubled. For disruption of rigid cell walls a mechanical cell treatment step was performed additionally by adding 500 µl of zirconia-silica beads (Ø 0.1 mm and 0.5 mm, mixed 1:1 (v/v)) (Carl Roth GmbH Co. KG, Karlsruhe, Germany) to the lysate followed by vigorously shaking in a bead beater (Retsch mill MM301; Retsch GMBH & CO.KG, Haan, Germany) during 2.5 min at a maximal frequency of 30 s^−1^ and afterwards allowed to cool on ice for 3 min. The mechanical cell disruption step was repeated before centrifugation of the assay during 1 min at 12,000 × g. The liquid supernatant, except the fat layer on top, was transferred to a column of the GenEluteTM Bacterial Genomic DNA Kit and DNA was further extracted as suggested by the manufacturer. The DNA concentration was determined using a ND-1000 Spectrophotometer (NanoDrop Technologies, Wilmington, USA).

For generation of 16S rDNA amplicons different primer sets were applied, covering the whole 16S rDNA sequence corresponding to position EC1–EC1510 of the *Escherichia coli* 16S rRNA gene [29], and leading to size fractions of approximately 450 bp, 550 bp or 650 bp length (Table 1). PCR was administered as described before in a 50 µl total volume using 53 ng metagenomic DNA and 25 pmol primers, each in a separate PCR reaction. Properties of the amplification steps were the following: Initial denaturation at 98 °C for 2 min, followed by 25 cycles, each of 10 s 98 °C, 15 s at the according annealing temperature listed in Table 1 for the according primers applied, 30 s 72 °C and a final extension at 72 °C during 5 min. Separate PCR products were pooled for generation of a sequencing library.

**Table 1.** Primers used for amplification of 16S rRNA genes.

Target	16S rRNA gene region	Primer name	Sequence (5′–3′)	Temp (°C)	References
*Bacteria*	V1–V3	TPU1	AGAGTTTGATCMTGGCTCAG	61	[30]–[33]
		U529R	ACCGCGGCKGCTGGC		
	V3–V4	Uni340F	CCTACGGGRBGCASCAG	56	[34]
		Uni806R	GGACTACNNGGGTATCTAAT		
	V4–V6	U515F	GTGCCAGCMGCCGCGGTAA	70	[30],[35]–[37]
		R1064	CGACRRCCATGCANCACCT		
	V5–V8	TPU4	GGATTAGATACCCTGGTAGTCC	63	[32],[38],[39]
		BS-R1407	GACGGGCGGTGWGTRC		
	V7–V9	BS-F1099	GYAACGAGCGCAACCC	56	[39]
		pB-00545	ACGGYTACCTTGTTACG		[40]
*Archaea*	V1–V3	A2Fa_minusGA	TTCCGGTTGATCCYGCCG	59	[41]
		U529R	ACCGCGGCKGCTGGC		[30],[33]
	V4–V6	U515F_minus5′base	TGCCAGCMGCCGCGGTAA	70	[30],[35]–[37]
		1048arcR-major	CGRCGGCCATGCACCWC		
		1048arcR-minor	CGRCRGCCATGYACCWC		
	V7–V8	A1040F-minusC	GAGAGGWGGTGCATGGC	63	[33],[42]
		UA1406R	ACGGGCGGTGWGTRCAA		

Amplified 16S rDNA amplicons were sequenced using GS FLX Titanium chemistry on a 454 Genome Sequencer FLX platform (Roche Diagnostics Ltd., Burgess Hill, West Sussex, United Kingdom) according to Roche 454 protocols. Sequences not passing the FLX quality controls were not considered for bioinformatics analysis, the 454-specific portions of the primers were trimmed, the raw sequences were sorted according to tag sequences and reads with low quality scores (quality score below 40) and short lengths (less than 200 bp) were removed. The resulting sequence reads were checked for chimera constructs using the program UCHIME based on the alignments to full length good quality and non-chimeric reference 16S rDNA sequences (downloaded from RDP) [43].

OTU assignment was performed using the RDP (Ribosomal Database Project) Classifier v2.7 [44] with taxonomic classification based on NCBI taxonomy (http://www.ncbi.nlm.nih.gov/taxonomy). OTUs represented by less than 5 reads were excluded from further analysis. The resulting OTUs were consolidated across different sampling points and normalized to percent counts using R statistical package.

### 2.4. Next generation sequencing of total RNA for taxonomic assignment of 16S rRNA transcripts

Total RNA was extracted from cheese surface smear samples applying the protocol described by Monnet and colleagues [45] and converted to cDNA. Subsequently the cDNA was sheared and the region corresponding to the 16S rRNA was selectively enriched by a set-up called vectorette [46], using the universal primer U515F as specific primer and 13 cycles of amplification. After size selection (about 600 bp) the libraries were sequenced using GS FLX Titanium chemistry on a 454 Genome Sequencer FLX platform (Roche Diagnostics Ltd., Burgess Hill, West Sussex, United Kingdom) according to Roche 454 protocols. Sequences not passing the FLX quality controls were not considered for bioinformatics analysis, the 454-specific portions of the primers were trimmed, the raw sequences were sorted according to tag sequences and reads with low quality scores (below 40) and short lengths (less than 200 bp) were removed. The resulting 16S rRNA reads were used for subsequent taxonomic classification of the metabolic active cheese surface microbiota as described previously for the sequencing of the 16S rDNA amplicons.

### 2.5. Nucleotide sequence accession numbers

The data from DNA and cDNA (RNA) sequencing are available through the NCBI GenBank Sequence Read Archive (SRA) database under the accession number SRP063818.

### 2.6. Statistical analysis

For visualization of the ordering of the cheese smear samples in reduced dimension (2D space) and for comparison between the technical approaches, principal components (PC) analysis was conducted on the correlation matrix calculated with JMP 10.0 software (SAS Institute AG, Wallisellen, Switzerland). Principal components for the first three dimensions were computed and the results for the first two principal components (PC) were visualized as PC plots. To evaluate the correlation between the applied microbial analysis techniques, results for DNA and RNA analysis were combined for each approach.

## 3. Results

### 3.1. Culture based analysis of microbial composition

**Figure 1.**
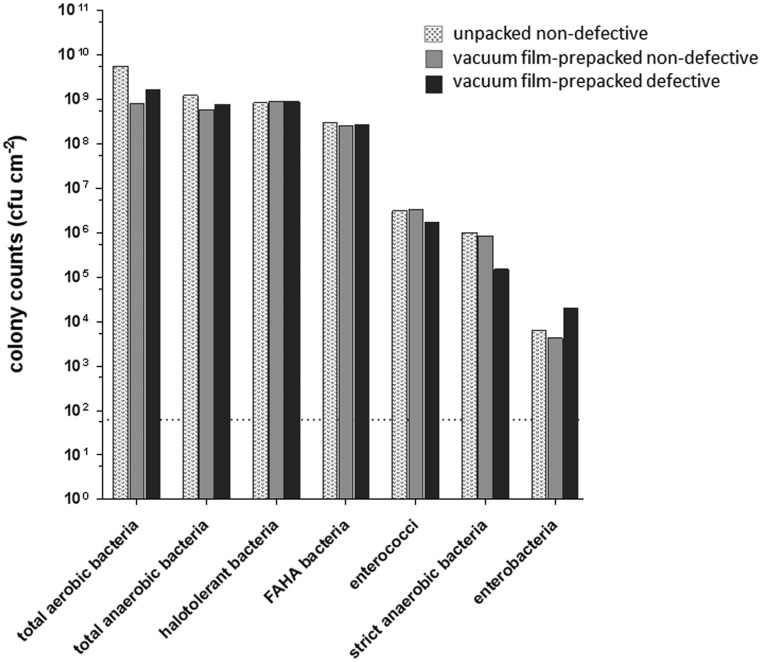
Plate count results are provided as weighted average in colony forming units (cfu cm^−2^) for total aerobic mesophilic bacteria, total anaerobic bacteria, halotolerant bacteria, facultative anaerobic halophilic and alcaliphilic (FAHA) bacteria, and enterobacteria for unpacked non-defective, vacuum film-prepacked non-defective and vacuum film-prepacked defective cheese smear microbiota. The detection limit is indicated by the dashed horizontal line.

Weighted average colony counts in cfu cm^−2^ were determined for unpacked, vacuum film-prepacked non-defective and vacuum film-prepacked defective cheese surface smear samples. The results from the culture based investigation and comparison of the microbial composition of the differing surface smear samples are provided in Figure 1. Counts for total mesophilic aerobic bacteria, total anaerobic bacteria, halotolerant bacteria and facultative anaerobic halophilic and alcaliphilic (FAHA) bacteria were in the range of 2.5 × 10^8^ to 5.5 × 10^9^ cfu cm^−2^ for all samples analyzed. Enterococci and strictly anaerobic bacteria revealed 1.5 × 10^5^ to 3.4 × 10^6^ cfu cm^−2^, enterobacteria ranged from 1.9 × 10^3^ to 2 × 10^4^ cfu cm^−2^ in all samples.

**Figure 2.**
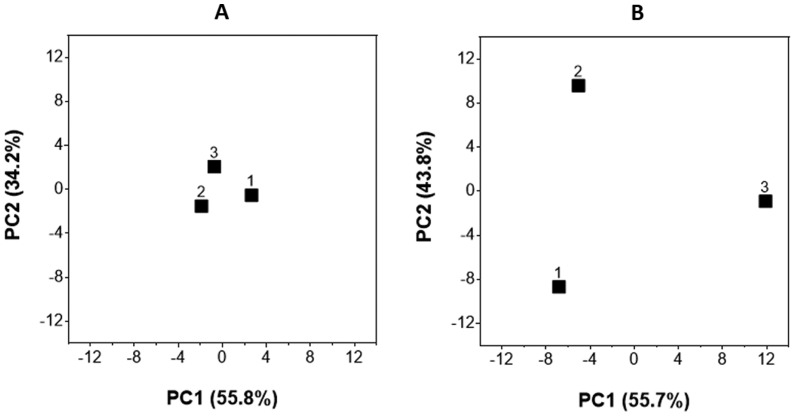
Principal coordinates analysis plots for comparison of the microbial composition determined by culture-based analysis (A) and next-generation sequencing analysis (B) of unpacked non-defective [1], vacuum film-prepacked non-defective [2] and vacuum film-prepacked defective [3] cheese surface smear samples. The percentage variation explained by the according principal component (PC) is indicated on the according x- or y-axis.

Overall, no substantial differences were detected that could be associated with vacuum film-prepackaging or a defective cheese surface smear. This becomes also obvious from the principal components analysis showing equidistant separation of the three sample types by the first two principal components explaining in total 90% of the data variation (Figure 2A).

### 3.2. Composition and diversity of the cheese surface microbiota analyzed by next-generation sequencing of amplified 16S rRNA gene fragments and total RNA

In order to perform the most comprehensive approach currently available for the investigation of complex microbial ecosystems, the microbial composition of the cheese surface smear was analyzed by next-generation sequencing of amplified 16S ribosomal RNA gene fragments. Furthermore, the proportion of metabolically active bacteria was investigated by high-throughput sequencing of total RNA followed by a taxonomic assignment of the obtained 16S rRNA reads.

The sequencing of amplified 16S rDNA fragments from the smear microbiota of vacuum film-prepacked cheese samples resulted in a total of 10,688 reads from defective and 10,780 reads from non-defective smear, respectively. For the sample from unpacked cheese 19,121 reads were generated. The number of reads that passed the quality check and were applied for taxonomic profiling was 14,413 reads for the sample from unpacked cheese. The sample from vacuum film-prepacked cheese with defective smear resulted in 7910 reads and 7708 reads were obtained from the sample of vacuum film-prepacked cheese with non-defective smear, respectively. With respect to total RNA sequencing 6361 to 17,952 total RNA reads were generated from vacuum film-prepacked cheese samples and 40,308 total RNA reads from the unpacked cheese sample. After quality check and restriction to reads originating from 16S rRNA the read numbers used for analysis were 2285 reads for vacuum film-prepacked cheese with defective smear, 2518 reads vacuum film-prepacked cheese with non-defective smear, and 2774 reads from unpacked cheese, respectively.

**Figure 3.**
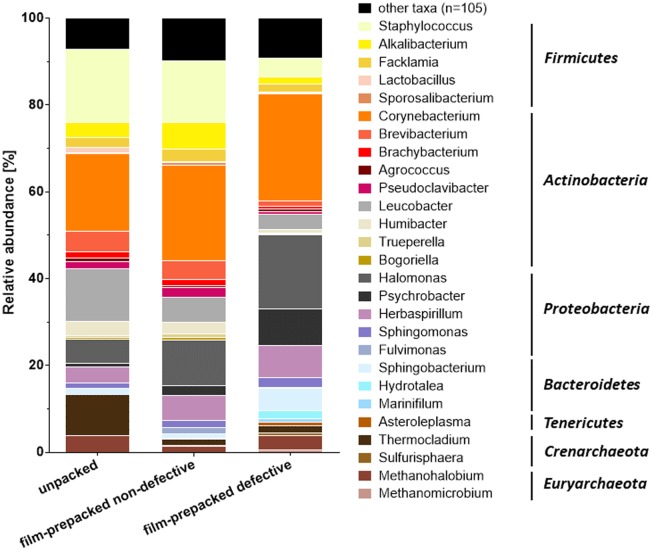
Comparison of the cheese smear microbiome of unpacked, vacuum film-prepacked non-defective and vacuum film-prepacked defective cheese smear samples determined by next-generation sequencing of 16S rDNA amplicons. Columns show the relative abundance (%) of prokaryotic genera detected in each sample. The most abundant phylotypes are displayed in different colors, while taxa revealing less than 0.5% of total reads are combined and shown in black.

**Figure 4.**
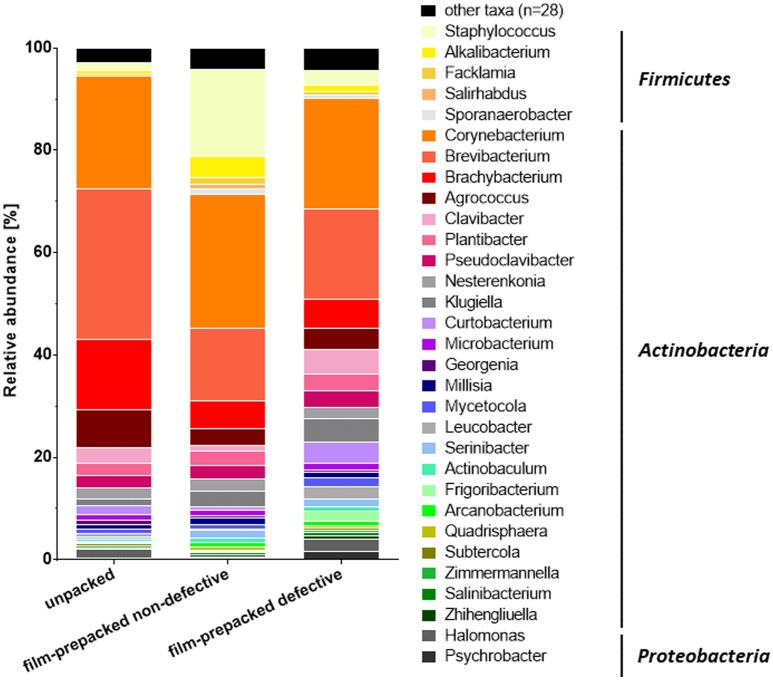
Analysis of the cheese smear microbiome of unpacked, vacuum film-prepacked non-defective and vacuum film-prepacked defective cheese smear samples performed by next-generation sequencing of total RNA extracted from smear samples. The columns display the relative abundance (%) of 16S rRNA reads for different prokaryotic genera within each cheese sample. The most abundant phylotypes are shown in different colors, taxa revealing less than 0.5% of total reads are combined and shown in black.

Sequencing of amplified 16S rDNA fragments revealed an extraordinarily diverse surface smear microbiota comprising 132 different OTUs (Figure 3), whereas the analysis of 16S rRNA reads resulted in only 59 different OTUs (Figure 4). However, 105 of the 132 OTUs detected by the 16S rDNA based approach revealed rather low relative abundance of less than 0.5% per sample (corresponding to less than 37 reads per sample). Therefore, they were summarized as “other taxa” in Figure 3 and Table S1. In the 16S rRNA based approach 28 of the 59 detected OTUs accounted for less than 0.5% relative abundance per sample (corresponding to less than 14 reads per sample) and were summarized as “other taxa” in Figure 4 and Table S2. Taxonomic assignment of the 16S rDNA reads on the genus level revealed that half of the reads belonged to facultative anaerobic bacteria, up to a third to strict aerobic bacteria and only 4 to 15% of the reads derived from strict anaerobic bacteria.

The 16S rRNA based analysis revealed up to 1% reads of strict anaerobic bacteria in the samples from vacuum film-prepacked cheese, whereas the unpacked cheese samples featured no strict anaerobes. The metabolically active smear community was primarily represented by aerobic *Actinobacteria* of the genera *Corynebacterium*, *Brevibacterium*, *Brachybacterium* and *Agrococcus*, which provided the largest numbers of 16S rRNA reads. Taken together they contributed to a relative abundance of 73% in the sample from unpacked cheese. *Corynebacterium* was also the most abundant taxon detected by the 16S rDNA based approach, whereas *Brevibacterium*, *Brachybacterium* and *Agrococcus* accounted for far less reads. Other *Actinobacteria* such as *Clavibacter*, *Plantibacter*, *Klugiella*, *Microbacterium* and *Leucobacter* constituted to a considerable amount of 19.75–37.11% relative abundance of 16S rRNA reads (Figure 4 and Table S2). *Staphylococcus* 16S rRNA reads represented a considerable proportion in the microbiota of the vacuum film-prepacked non-defective cheese sample, while this was not the case for the other smear samples. However, in the 16S rDNA based analysis, reads from *Firmicutes* were far more prominent in all three smear sample types with 8.10–23.90% relative abundance. Whereas *Proteobacteria* revealed up to 35.20% relative abundance and were represented by diverse genera such as *Halomonas*, *Psychrobacter*, *Herbaspirillum*, *Sphingomonas* and *Fulvimonas* in the rDNA based approach, they were far less represented by only few reads from *Halomonas* and *Psychrobacter* in the 16S rRNA based approach. *Archaea* were in principle represented by 16S rDNA reads (3–12% relative abundance) from the genera *Thermocladium*, *Sulfurisphaera*, *Methanohalobium* and *Methanomicrobium* as 16S rRNA reads from *Archaea* revealed less than 0.5% relative abundance. The most striking difference in the composition of the microbial community structure in the smear from unpacked, vacuum film-prepacked non-defective and vacuum film-prepacked defective cheese was the strong increase in populations of gram-negative *Proteobacteria* and *Bacteroidetes*, in particular for the sample from defective smear. The abundance of gram-positive staphylococci, which belong to the typical smear microbiota, revealed a pronounced decrease in the smear of vacuum film-prepacked defective cheese.

Overall, the differences in the microbial community structure determined by comparison of the results from the 16S rDNA and 16S rRNA based approach indicated that the majority of ribosomal RNAs derived from a few taxa which represent the metabolically active part of the cheese smear microbiota. When the results of sequencing 16S rDNA and 16S rRNA were considered separately in principal components analysis, the first principal component clearly clustered the individual samples according to the target molecules (data not shown). However, when the entire high-throughput sequencing data per smear microbiota was taken into account, the first two principal components, which explained in total 99.48% of the variation, equidistantly separated the microbiota of the unpacked, vacuum film-prepacked non-defective and vacuum film-prepacked defective cheese smear (Figure 2).

Furthermore, it shows that the different approaches applied for analysis of the surface smear microbiota provided corresponding results. The low resolution of culture-based analysis revealed a less different community structure of unpacked, vacuum film-prepacked non-defective and vacuum film-prepacked defective cheese smear samples (Figure 2A). Concurrent with the highest resolution at a global scale, high-throughput sequencing analysis resulted in greatest differences in microbial community composition of the different smear samples (Figure 2B). In summary, the applied next-generation sequencing approach allowed an in-depth analysis of the cheese smear microbiota. Whereas the detection of 16S rDNA reads demonstrated the presence of a broad hitherto unknown spectrum of specific microbial taxa, the obtained 16S rRNA reads verified the metabolic activity of a certain subset of aerobic and facultative anaerobic *Actinobacteria* mainly represented by typical cheese smear bacteria.

## 4. Discussion

Traditional culture-based analyses of complex microbiota in food systems are limited in their ability to provide a profound insight into the actual microbial composition and are strongly biased by the selected cultivation media and the cultivability of microorganisms [47]. As a faster and more accurate method providing higher specificity and sensitivity, molecular community fingerprint techniques have been state-of-the-art techniques to study the microbial diversity of complex ecosystems before they have been replaced by next-generation sequencing technologies for high-throughput purposes [20],[48]. Previous studies based on next-generation sequencing of cheese microbiota mainly focused on the analysis of individual amplified “hypervariable regions” of 16S rRNA genes [49]–[54]. In this study, the cheese surface microbiome was analyzed by a next-generation sequencing approach considering not only the different “hypervariable regions” V1–V9 of the 16S ribosomal RNA gene, but also the direct sequencing of total RNA without prior amplification. Thus, in addition to the composition and diversity, also the metabolic active proportion of the cheese surface microbiota was analyzed for the different samples from unpacked, vacuum film-prepacked non-defective and vacuum film-prepacked defective cheese. It is a preliminary study carried out on just one cheese batch produced on the same day and ripened in the same cheese cellar under identical conditions, which needs further investigation in the future to improve our knowledge on the impact of different packaging strategies on the cheese smear microbiome.

The microbial community patterns obtained from the different samples revealed an altered composition primarily caused by an increase in populations of gram-negative *Proteobacteria* and *Bacteroidetes* and a decrease in staphylococci, which are part of the typical smear microbiota. These alterations were in particular pronounced in the samples of vacuum film-prepacked cheese showing defective smear. This observation is contrary to data from culture-based investigation of the cheese surface smear defect, which did not reveal distinct differences between the different sample types. A previous study on red-smear cheese defect development [25] the stable microbial composition determined during the cheese storage period indicated a complex etiology, where more than a single factor contributes to the smear defect development. In a later study, it was shown that factors such as anaerobic conditions, an increase in water activity as well as a decrease in pH was correlated with a diminished rRNA transcriptional activity of the smear microbiota [26]. In the present study, the smear microbiota of vacuum film-prepacked cheese samples featured considerably less 16S rRNA reads and the composition of the microbial community structure appeared to be less affected compared to the 16S rDNA based next-generation sequencing approach, which revealed clear changes in the microbial population structures of the different samples.

In particular, reads from microorganisms typically for the surface of smear-ripened cheese, such as *Corynebacterium*, *Staphylococcus*, *Brevibacterium*, *Arthrobacter*, *Micrococcus*, *Microbacterium*, *Alkalibacterium* and *Facklamia* predominated the results of the 16S rDNA based analysis. With the exception of *Micrococcus* most of the bacteria typical for a smear cheese surface have been isolated from the surface of the same cheese variety in a previous study [25]. In this culture based study typical smear bacteria represented the major part of the smear microbiota with colony counts of 10^7^ to 10^8^ cfu cm^−2^. This was also the case in the present study were *Corynebacterium*, *Brevibacterium* and *Brachybacterium* accounted for the major part of identified 16S rDNA reads. This finding seems reasonable because representatives of these genera are known to contribute substantially to the typical smear-cheese characteristics during the ripening process by production of extracellular proteinases and lipases, pigments and aroma compounds [4],[8],[55],[56]. Due to the known application in starter cultures for cheese production the 16S rDNA reads from *Lactobacillus* detected in this study might have derived from bacteria of the cheese matrix [4],[57].

Besides microorganisms already known to predominate on the cheese surface, the surface smear microbiota was found to comprise an unexpected high microbial diversity implying many taxa hitherto not related to the cheese ecosystem. As the sampled cheeses were produced from raw milk and a high biodiversity of indigenous microorganisms is characteristic for raw milk, a major part of the cheese smear microbiota might derive from this source [57],[58]. Comparison of the genera represented by 16S rDNA and 16S rRNA sequences with scientific literature revealed manifold relations of initially unexpected taxa to the farm environment, such as the bovine microbiota, but also further relations to water, marine and salty ecosystems or soil. The genus *Trueperella* is known to be common in cow milk and predominates mainly bovine mastitis milk [54],[58]–[65], while *Asteroleplasma* is part of the cattle rumen ecosystem [66],[67]. *Herbaspirillum* is a genus, which is associated with roots of herbaceous plants, while *Humibacter* and *Fulvimonas* are related to soil or compost [68]–[71]. It is known that farm related microorganisms can also be transferred to cheese via raw milk used for cheese production, while salt crystals can serve as a source for microorganisms when cheese samples pass a brine bath during cheese production or are regularly smeared with brine during the ripening process. Furthermore, the 16S rDNA based approach revealed the presence of *Archaea* in the cheese smear microbiota. However, rDNA reads from *Archaea* were rare, and 16S rRNA reads accounted for less than 0.5% of all reads. The 16S rDNA based approach revealed *Thermocladium*, a taxon recently detected by pyrosequencing in buttermilk, and *Methanomicrobium*, which occurs naturally in bovine rumen. *Sulfurisphaera* and *Methanohalobium* are known to inhabit marine water systems or saline lagoons [72]–[75].

Because RNA is less stable than DNA and its expression is tightly related to the bacterial physiological status, ribosomal 16S RNA represents an appropriate marker for metabolically active and integer cells [76]–[80]. Thus, the detected 16S rRNA reads represent the composition of the metabolic active bacteria in the cheese surface smear. Consequently, the abundance of 16S rRNA reads from *Actinobacteria* indicates that this phylum represents the largest population of active bacteria on the cheese surface post ripening. Furthermore, *Agrococcus* provided a considerable number of the 16S rRNA reads in the functional communities analyzed. Although usually not numbered among the typical smear microorganisms, *Agrococcus* was previously detected in cheese smear and identified as a member of the “house microbiota” in dairies. Moreover, the first isolation of *Agrococcus casei* has been described for the surface of Gubbeen, Livarot and Tilsit cheese [11],[81]–[83].

Besides *Corynebacterium*, *Brevibacterium*, *Brachybacterium* and *Agrococcus*, which predominated the metabolic active community on the cheese surface, a variety of other genera from *Actinobacteria* contributed to a considerable proportion of the 16S rRNA reads. *Microbacterium* spp. are considered to belong to the typical smear bacteria, which frequently colonize the cheese surface in high numbers of 10^8^ cfu cm^−2^ and are usually part of the “house microbiota” in cheese dairies. Representatives of *Curtobacterium*, *Klugiella*, *Pseudoclavibacter*, *Leucobacter*, and *Mycetocola* were occasionally described in cheese surface smear or cheese dairy and farm environment [11],[15],[56],[58],[59],[61],[63],[84]–[88]. Although *Clavibacter*, *Plantibacter*, *Actinobaculum*, *Frigoribacterium*, *Zimmermannella*, *Arcanobacterium* and *Subtercola* have so far not been described to inhabit cheese surface smear, recent studies indicate that members of these genera play a role in the context of milk, farm and dairy animal ecosystems [58],[61],[63],[87],[89]–[93]. Further studies indicate that raw milk may serve as a vector for the genera *Georgenia*, *Millisia* and *Quadrisphaera*, which usually inhabit terrestrial natural ecosystems such as soil or sludge [82],[94],[95]. The application of salt during the cheese production process is suggested as intrinsic source for microorganisms such as *Salirhabdus*, *Nesterenkonia*, *Serinibacter*, *Salinibacterium* and *Zhihengliuella*, which are typically isolated from marine or saline aquatic ecosystems [96]–[99]. The actual role and contribution by the various unexpected microorganisms occurring on the cheese surface remains unknown, but the previously cited literature revealed production of lipases, proteases, pigments and chemical aroma compounds or the coagulation of milk by various of these *Actinobacteria*.

The comparison of 16S rDNA results for samples showing varying cheese smear quality revealed a decrease of reads from staphylococci in the samples from defective smear, in particular when compared with the sample from unpacked cheese. Staphylococci represent typical cheese smear microorganisms which are known to predominate the cheese surface smear in particular at the beginning of the ripening process, providing conditions for better growth of corynebacteria [12],[100]. Thus, their decline suggests unfavorable conditions in defective smear. Another striking observation was the higher abundance of representatives from *Proteobacteria* and *Bacteroidetes* in the smear of prepacked cheese, which was most pronounced in samples from defective smear. The phylum *Bacteroidetes* was mainly represented by reads from *Sphingobacterium* and *Hydrotalea*, which are known to be abundant in soil and water [101],[102], but have not been associated with cheese production environment so far. The *Proteobacteria* were primarily represented by reads from the genera *Psychrobacter* and *Halomonas*, which have been frequently detected in high numbers in the surface smear of red-smear cheese varieties in previous studies [54],[103],[104]. Usually, high cell counts of *Proteobacteria* during the early stages of the ripening period decrease with time, due to the combined effects of different physical and chemical parameters [105]. For *Psychrobacter celer* it has been shown that the bacteria are able to successfully implant itself in cheese, regardless of its inoculation level. They contributed to the production of volatile aroma compounds such as aldehydes, ketones and sulfur compounds and had an impact on the aromatic properties of the cheese [106]. This observation is supported by whole genome sequencing of a *Psychrobacter* strain isolated from cheese rind, which revealed that the genome harbors enzymes that are important for cheese ripening in other bacteria, such as cystathionine/methionine beta or gamma-lyases, many proteases and peptidases, aminotransferases, and lipases [107]. Due to these properties increased levels of the bacteria may contribute to the unpleasant off-flavor that accumulates under the foil of prepacked smear cheese. For *Halomonas venusta* it was shown that the bacteria produce cadaverine in a cheese model for production of volatile compounds as well as biogenic amines [108]. However, *Halomonas* spp. are better known as potent exopolysaccharide producers [109],[110]. This property may potentially contribute to the sticky and slimy cheese surface of foil prepacked smear cheese. Thus, the combination of a decrease of typical smear organisms like staphylococci, together with an increase of members of *Proteobacteria* in defective smear might substantially contribute to the observed negative organoleptic properties of the surface smear of cheese after prepacking in plastic foil.

## 5. Conclusions

Overall, the data obtained by the high-throughput sequencing approach revealed an unexpectedly high overall diversity in the cheese smear microbiota and comprised many bacteria that have hitherto not been described for the cheese ecosystem. However, all of the detected taxa bear relation to the farm or cheese dairy environment as shown in literature. Although the next-generation sequencing approach applied in this study provides currently the most detailed microbiome analysis, a future challenge would be to trace the source of the microorganisms colonizing the surface of smear-ripened cheeses, and to monitor the microbial succession during the cheese ripening process. Therefore, a polyphasic approach by combining different molecular techniques with culture-based methods should be considered to study complex microbial ecosystems such as cheese smear. Moreover, for a better understanding of the microbial contribution to cheese ripening and for unraveling the function of certain microbial populations, a thorough analysis of the cheese smear metatranscriptome would be an indispensable prerequisite.
