# Supplementary material for: The role of the surface smear microbiome in the development of defective smear on surface-ripened red-smear cheese
Source: AIMS Microbiol. 2018 Oct 25;4(4):622–41. doi: 10.3934/microbiol.2018.4.622 (PMC6613336; doi:10.3934/microbiol.2018.4.622)
Supplement: Supplementary file 1 [file microbiol-04-04-622-s001.pdf]

AIMS Microbiology

AIMS Microbiology, 4(4): 622–641.
DOI: 10.3934/microbiol.2018.4.622
Received: 29 June 2018
Accepted: 20 September 2018
Published: 25 October 2018

http://www.aimspress.com/journal/microbiology

Research article

The role of the surface smear microbiome in the development of defective smear on surface-ripened red-smear cheese

Jasmine S. Ritschard[1,]†, Lea Amato[2,]†, Yadhu Kumar[3], Britta Müller[3], Leo Meile[2] and Markus Schuppler[1,]*

[1] Laboratory of Food Microbiology, Institute of Food, Nutrition and Health, ETH Zurich, Schmelzbergstrasse 7, 8092 Zurich, Switzerland
[2] Laboratory of Food Biotechnology, Institute of Food, Nutrition and Health, ETH Zurich, Schmelzbergstrasse 7, 8092 Zurich, Switzerland
[3] Eurofins GATC Biotech AG, Jakob-Stadler-Platz 7, 78467 Konstanz, Germany

* **Correspondence:** Email: markus.schuppler@hest.ethz.ch; Tel: +41446327862; Fax: +41446321266.

† These two authors contributed equally.

Supplementary

Table S1. Related to Figure 3. Relative abundances of 16S rDNA amplicons determined by next-generation sequencing of cheese smear samples.

Phylum	Genus	Unpacked<br>14413 reads	Vacuum film-prepacked non-defective<br>7708 reads	Vacuum film-prepacked defective<br>7910 reads
		Relative abundance (%)		
		rDNA	rDNA	rDNA
	other taxa (n = 105)[a]	7.19	9.80	9.27
Firmicutes	*Staphylococcus*	16.83	14.22	4.20
	Alkalibacterium	3.50	6.23	1.64
	Facklamia	2.14	2.71	1.98
	Lactobacillus	1.42	0.19	0.09
	Sporosalibacterium	0.07	0.65	0.21
Actinobacteria	*Corynebacterium*	17.92	21.95	24.73
	Brevibacterium	4.74	4.46	1.18
	Brachybacterium	1.33	1.50	0.63
	Agrococcus	0.95	0.40	0.52
	Pseudoclavibacter	1.67	2.11	0.68
	Leucobacter	12.13	5.76	3.55
	Humibacter	3.17	2.74	0.73
	Trueperella	0.42	0.80	0.33
	Bogoriella	0.44	0.54	0.25
Proteobacteria	*Halomonas*	5.63	10.56	16.92
	Psychrobacter	0.86	2.26	8.53
	Herbaspirillum	3.58	5.70	7.40
	Sphingomonas	1.28	1.80	2.25
	Fulvimonas	0.03	1.35	0.10
Bacteroidetes	*Sphingobacterium*	1.37	1.06	5.27
	Hydrotalea	0.00	0.00	1.73
	Marinifilum	0.00	0.09	0.86
Tenericutes	*Asteroleplasma*	0.00	0.00	0.75
Crenarchaeota	*Thermocladium*	9.37	1.60	1.74
	Sulfurisphaera	0.17	0.09	0.63
Euryarchaeota	*Methanohalobium*	3.75	1.41	3.26
	Methanomicrobium	0.03	0.00	0.56

[a]Summary of the relative abundances of taxa revealing less than 0.5% of total reads in each of the smear samples analyzed.

Table S2. Related to Figure 4. Relative abundances of 16S rRNA reads determined by next-generation sequencing of cheese smear samples.

Phylum	Genus	Unpacked	Vacuum film-prepacked non-defective	Vacuum film-prepacked defective
		2774 reads	2518 reads	2285 reads
		Relative abundance (%)		
		rRNA	rRNA	rRNA
	other taxa (n = 28)[a]	2.81	4.13	4.38
Firmicutes	*Staphylococcus*	1.66	17.00	2.84
	Alkalibacterium	0.65	4.13	1.36
	Facklamia	0.43	1.43	0.70
	Salirhabdus	0.00	0.75	0.00
	Sporanaerobacter	0.00	1.19	0.48
Actinobacteria	*Corynebacterium*	22.06	26.25	21.66
	Brevibacterium	29.45	14.18	17.77
	Brachybacterium	13.70	5.36	5.60
	Agrococcus	7.43	3.22	4.03
	Clavibacter	2.92	1.15	4.81
	Plantibacter	2.52	2.86	3.28
	Pseudoclavibacter	2.34	2.58	3.33
	Nesterenkonia	2.09	2.50	2.28
	Klugiella	1.33	3.02	4.42
	Curtobacterium	1.80	0.56	4.29
	Microbacterium	1.01	1.07	1.18
	Georgenia	0.87	0.44	0.61
	Millisia	0.94	1.31	1.05
	Mycetocola	0.87	0.91	1.62
	Leucobacter	0.61	0.24	2.36
	Serinibacter	0.47	1.55	1.53
	Actinobaculum	0.43	0.75	1.01
	Frigoribacterium	0.25	0.00	1.97
	Arcanobacterium	0.00	0.83	0.83
	Quadrisphaera	0.25	0.71	0.48
	Subtercola	0.50	0.24	0.35
	Zimmermannella	0.36	0.32	0.53
	Salinibacterium	0.18	0.28	0.70
	Zhihengliuella	0.00	0.52	0.48
Proteobacteria	*Halomonas*	1.77	0.32	2.41
	Psychrobacter	0.29	0.20	1.66

[a]Summary of the relative abundances of taxa revealing less than 0.5% of total reads in each of the smear samples analyzed.

AIMS Press

© 2018 the Author(s), licensee AIMS Press. This is an open access article distributed under the terms of the Creative Commons Attribution License (http://creativecommons.org/licenses/by/4.0)
